# Growth and Survival of *Mesorhizobium loti* Inside *Acanthamoeba* Enhanced Its Ability to Develop More Nodules on *Lotus corniculatus*

**DOI:** 10.1007/s00248-015-0587-6

**Published:** 2015-03-17

**Authors:** Magdalena A. Karaś, Anna Turska-Szewczuk, Dominika Trapska, Teresa Urbanik-Sypniewska

**Affiliations:** Department of Genetics and Microbiology, Maria Curie-Sklodowska University, Akademicka 19, 20-033 Lublin, Poland

**Keywords:** *Mesorhizobium*, Endosymbiosis, *Acanthamoeba*, Coculture

## Abstract

The importance of protozoa as environmental reservoirs of pathogens is well recognized, while their impact on survival and symbiotic properties of rhizobia has not been explored. The possible survival of free-living rhizobia inside amoebae could influence bacterial abundance in the rhizosphere of legume plants and the nodulation competitiveness of microsymbionts. Two well-characterized strains of *Mesorhizobium*: *Mesorhizobium loti* NZP2213 and *Mesorhizobium huakuii* symbiovar *loti* MAFF303099 were assayed for their growth ability within the Neff strain of *Acanthamoeba castellanii*. Although the association ability and the initial uptake rate of both strains were similar, recovery of viable *M. huakuii* MAFF303099 after 4 h postinfection decreased markedly and that of *M. loti* NZP2213 increased. The latter strain was also able to survive prolonged co-incubation within amoebae and to self-release from the amoeba cell. The temperature 28 °C and PBS were established as optimal for the uptake of *Mesorhizobium* by amoebae. The internalization of mesorhizobia was mediated by the mannose-dependent receptor. *M. loti* NZP2213 bacteria released from amoebae developed 1.5 times more nodules on *Lotus corniculatus* than bacteria cultivated in an amoebae-free medium.

## Introduction

Rhizobia are soil bacteria that colonize legume roots and induce nodules in which atmospheric nitrogen is converted into plant-available compounds. The number and diversity of indigenous rhizobia in the rhizosphere depends on a number of abiotic and biotic factors [1–3] and proximity to other organisms [4]. The impact of protozoan grazing on microbial communities has been reported as one of the mechanisms controlling bacterial populations in soil [5, 6]. Not all bacteria are an equally suitable food source for protozoa, and it was demonstrated that some, mostly Gram-negative bacteria, were able to survive in the vicinity of protozoa [7].

*Acanthamoeba castellanii* is a ubiquitous free-living amoeba (FLA) found in a wide range of environments. This organism is resistant to wide temperature ranges, desiccation and disinfection [8]. *A. castellanii* can also be the cause of life-threatening granulomatous amoebic encephalitis (GAE) or infection of the cornea, i.e. amoebic keratitis, a more common disease occurring in people. It has been reported that around 25 % of *Acanthamoeba* isolates recovered from clinical and environmental sources harbour intracellular pathogenic bacteria, viruses or fungi [7, 9]. Among amoeba-associated microorganisms (AAM), a number of rhizobial species have been identified. *Mesorhizobium amorphae* and *Bradyrhiozbium* spp. were isolated in coculture with *Acanthamoeba polyphaga* from human nasal swabs and hospital water network tanks [10–12]. Thomas et al. [13] showed that *Bradyrhizobium japonicum*, a symbiont of soybean (*Glycine max*), was able to survive inside *Hartmannella* spp. and was classified into amoeba-resistant bacteria (ARB). The strain ‘*Candidatus* Bradyrhizobium massiliensis’ closely related to *B. japonicum* (97 % of 16S rRNA gene similarity) led to *A. polyphaga* cell lysis after 5 days of coculture [14]. Conversely, in the case of *Sinorhizobium meliloti*, a large number of bacteria were consumed by *Hartmannella* spp., *Naegleria* spp. and *Vahlkampfia* spp. [5]. Thus, interactions between rhizobia and amoebae can lead to different implications depending on used systems.

Over the last 10 years, there has been an increasing interest in the study of bacterial endosymbiosis since the interactions between bacteria and amoebae may result in changes in the physiology of the bacteria, e.g. their enhanced virulence, increased susceptibility to antibiotics and resuscitation from a viable but non-culturable state [15–17]. Moreover, the growth inside amoebae is hypothesized to prepare the way for bacterial adaptation to mammalian cells [18]. Microbial pathogens improve their ability to persist facultatively in amoeba cells by competing with their defence system. Several steps are involved in the establishment of the endosymbiosis including the induction of bacterial secretion systems. Many ARB [7] express either the type III or the type IV secretion systems (T3SS and T4SS, respectively), which were determined to play a crucial role in the invasion and intracellular survival within amoeba cells [19, 20]. The effector proteins delivered by the T3SS and/or T4SS expressed by intracellular bacteria [20] can modulate the host cell response to its own advantage, thereby promoting survival of the bacteria. Distinct course of events takes place for interactions of amoeba—extracellular bacteria possessing T3SS (e.g. *Pseudomonas aeruginosa*). The secretion of effector proteins of T3SS leads to lysis of amoebal cells [21].

The T3SS, previously thought to be unique to pathogenic bacteria, has been identified in a number of rhizobia including *Rhizobium* sp. strain NGR234, *Bradyrhizobium diazoefficiens* USDA 110 (formely *B. japonicum* USDA 110), *Sinorhizobium fredii* USDA 257 and *Mesorhizobium huakuii* symbiovar *loti* MAFF303099, whereas the T4SS system has been identified in *M. loti* strains [22]. Both systems have been found to be obligate in the symbiotic relationships with the host plants. It was shown that the components of the T3SS system exerted either a positive or negative effect on the establishment of nitrogen-fixing symbioses with leguminous hosts [22, 23].

The narrow host-range rhizobia associated with *Lotus corniculatus* and other *Lotus* species were initially classified as *Rhizobium loti*. Later, when the genus *Mesorhizobium* was created, *R. loti* was reclassified as *Mesorhizobium loti*, which is considered the type species [24]. The ‘meso-growing’ rhizobia classified in the species *M. loti* were heterogeneous. For instance, strain MAFF303099 isolated from the root nodules of *L. japonicus* was switched to the species *M. huakuii* symbiovar *loti* based on the phylogenetic analyses and genome sequencing [25–27].

Since the biological nitrogen fixation will cause reduced use of nitrogen-based fertilizers, many efforts are undertaken to develop rhizobial inoculant strains [28]. In this context, it is important to study numerous traits affecting rhizobial competitiveness and their relationships with other organisms in the rhizosphere of the legume plants. In the present study, two well-characterized strains of *Mesorhizobium* spp.: *M. loti* NZP2213 and *M. huakuii* sbv. *loti* MAFF303099 (*Mesorhizobium* sp. MAFF303099) were used to investigate their interactions with *A. castellanii*. The ability of these bacteria to survive inside amoebae and the capability of rhizobia exposed to the amoebic intracellular environment to infect the root hairs of *L. corniculatus* were examined.

## Materials and Methods

### Strains of *Mesorhizobium* and Growth Conditions

The *Mesorhizobium loti* NZP2213^T^ (ATCC33669) strains, hereafter designated as NZP2213, [24] and *M. huakuii* symbiovar *loti* MAFF303099, hereafter designated as MAFF303099) [29], were used. These strains form a tight cluster with *M. loti* and *M. huakuii* strains [30]. One of the differences between the two strains is the presence of a T3SS gene cluster in MAFF303099 [29] and T4SS genes in the NZP2213 VirB/day-type [22, 23].

The strains were grown overnight in a liquid trypton-yeast extract medium (TY) on a rotary shaker (120 rev/min) at 28 °C for 24 h. Then, bacterial cells were harvested, washed three times with phosphate-buffered saline (PBS), pH 7.4, or a proteose yeast glucose medium (PYG), and the turbidity of the suspension was adjusted to the McFarland standard of 1.

### Culture of *Acanthamoeba*

The nonpathogenic *Acanthamoeba castellanii* Neff strain (ATCC 30010) and *Acanthamoeba* spp. strain P3, a clinical isolate from a keratitis patient and a kind gift from Prof. E. Hadaś (Poznan University of Medical Sciences, Poland), were used in cocultures. The amoebae were grown in 300 mL conical flasks in the PYG medium with shaking as described previously [31, 32]. The amoeba cultures were centrifuged at 100×*g* for 5 min; the sediments were washed three times with PBS and finally resuspended in PBS or PYG to a density of 6 × 10^5^ cells mL^−1^.

### Association Assays

The experiments were carried out according to the Alsam et al. [33] method with minor modification. The suspension of amoebae (1 mL) prepared as described above was placed in plastics tubes (10 mm internal diameter) and allowed to adhere for 24 h at 28 °C. Twenty-microlitre portions containing 6 × 10^6^ cells mL^−1^ of 24-h bacterial cultures suspended in PBS or PYG were used to seed the amoeba monolayers with multiplicity of infection (MOI) of 10. The tubes were centrifuged (1500×*g* for 30 min) to enhance the contact between bacteria and amoebae, and the incubation was carried out for 1 h at 18 °C or at 28 °C. Following the incubation, the extracellular medium was aspirated, and the amoebae layer was washed three times with PBS to remove non-adherent bacteria. At the final wash, the supernatant was plated onto TY agar to determine the presence of unbound bacteria. Finally, the number of amoeba cells was counted using a haemocytometer. The amoeba were lysed by sonication in an ultrasonic bath at 20–40 kHz for 2 min, and 10-fold dilutions of the lysates were plated onto TY agar to count the number of viable bacteria. The CFU values were enumerated after 3 days of incubation at 28 °C. The percentage of bacterial association was calculated as follows: recovered bacterial CFU/total bacterial CFU × 100 = % of bacterial CFU associated with *Acanthamoeba*. In addition, the ratio of bacteria to amoebae (the ratio of bacterial invasion/uptake) was calculated as follows: recovered bacterial CFU/number of *Acanthamoeba* cells = bacteria/*Acanthamoeba* ratio.

### Invasion Assays

The ability of bacteria to invade or to be taken up by *A. castellanii* was studied by a modified method of Alsam et al. [33]. The invasion assays were carried out similarly to the association assays except the measurement of CFU which was performed after 2-, 3- and 4-h co-incubation. After a given period, the amoebas were washed three times with PBS to remove most of the extracellular bacteria. The residual bacteria were killed by streptomycin and ampicillin (each at a concentration of 100 μg mL^−1^) during 24-h incubation followed by triple washing of amoebae with PBS. The final PBS wash was plated onto TY agar to ensure that any remaining extracellular bacteria had been killed. Finally, the amoebae were counted and ruptured by sonication in an ultrasonic bath (at 20–40 kHz, for 2 min), and the number of intracellular bacteria was determined as described above. Simultaneously, the sensitivity of the bacteria to streptomycin and ampicillin was tested.

Possible involvement of amoeba lectins in recognition of rhizobia was examined. The ability of exogenously added mannose, glucose and galactose to interfere with the bacterial invasion/uptake process was studied in the assays performed similarly to the invasion assays, i.e. 4-h co-incubation in PBS at 28 °C. In these experiments, prior to inoculation with the bacteria, the amoebae were pretreated with a solution of individual sugars (D-glucose, D-galactose, D-mannose) at a final concentration of 100 mM for 1 h at 28 °C followed by triple washing with PBS and centrifugation (at 100×*g* for 5 min) to remove unbound sugars.

To study if the signalling traits are triggered upon the uptake of *Mesorhizobium* bacteria, the amoeba were incubated with genistein (a protein tyrosine kinase inhibitor) at a final concentration of 50 and 100 μM in dimethylsulfoxid (DMSO) for 1 h at 28 °C. After the treatment, the amoeba suspensions were centrifuged (three times at 100×*g*, for 5 min each), the supernatant was discarded and the amoeba pellet was triple washed with PBS. Subsequently, the bacterial cultures were added, and the invasion assays were performed. The test was done to control the effect of the final DMSO concentration on amoebas.

### Intracellular Survival Assay and Release of Bacteria from Amoebae

To determine the effects of long-term cocultures of *Mesorhizobium* and *Acanthamoeba*, the survival assays were performed using a modified method of Alsam et al. [33]. The incubation time of amoebae with bacteria was 2 h. After removal of the bacteria by exposure to antibiotics followed by three times washing with PBS (this time was designated as 0 h postinfection), the incubation was prolonged to 24 h at 28 °C. The lysates of amoeba were plated on TY agar, and the bacterial colonies were counted.

The capability of the bacteria to self-release from the amoebae was tested after 4 h of the cocultivation. Afterwards, the amoebae with possibly internalized bacteria were treated with antibiotics and washed with PBS three times. The control of bacterial absorption was performed by seeding the amoeba without prior lysis on TY agar plates and incubation at 18, 28 and 37 °C.

### Plant Tests

Birdsfoot trefoil (*Lotus corniculatus* L. cv. Skrzeszowicka) seeds were surface sterilized and germinated at 28 °C on a nitrogen-free medium as described earlier [34]. Two-day-old seedlings were planted in nitrogen-free slants (one per tube) and allowed to grow for 7 days before being inoculated with 1 mL suspension (10^4^ CFU mL^−1^) of each strain. The plants were grown in a greenhouse under natural light supplemented with an artificial light regime (14/10 h light/dark). After 4 weeks, the plants were harvested, and the fresh mass of shoots and roots and the number of nodules were examined. Twenty seedlings and the uninoculated controls were used for each experiment.

### Statistical Analysis

All experiments were performed independently three times and each experiment comprised two samples. The values obtained were submitted to statistical analyses performed with GraphPad Prism 6.0 software and presented as means (±SD) of three independent experiments. The significance level of *p* < 0.05 was established using one-way analysis of variance (ANOVA) and Tukey’s test. The unpaired *t* test was applied for data that did not meet the assumptions of ANOVA.

## Results

### The Association/Internalization of *Mesorhizobium* Strains to *Acanthamoeba* sp.

To examine the character of the interaction between rhizobia (association and facultative invasion) and *Acanthamoeba*, the moderately growing strains of *Mesorhizobium*: *M. huakuii* sbv. *loti* MAFF303099 and *M. loti* NZP2213 were used.

**Fig. 1:**
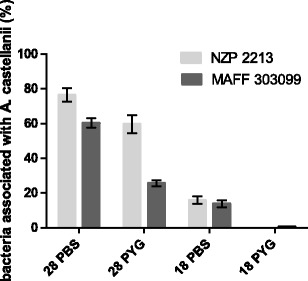
A level of binding/association of *Mesorhizobium* spp. with *A. castellanii* Neff presented as a percentage of bacterial inocula (CFU = 6 × 10^6^) vs. temperature (28 and 18 °C) and medium type (PBS vs. PYG). Results are the mean of three independent experiments performed in duplicate. *Error bars* represent standard deviation (*n* = 3)

The association assays were performed to determine the ability of the bacteria to interact with *A. castellanii*. Here, the term ‘association’ is used to describe both the rhizobia that were inside the amoebae and those that were attached on the surface of *Acanthamoeba*. The influence of nutrients and temperature on these relationships was studied (Fig. 1). The results obtained indicated that at 28 °C, the rates of association of NZP2213 to *A. castellanii* were similar in PBS and in the PYG medium (76.44 % ± 3.9 and 59.86 % ± 5.3, respectively). The rate of association of strain MAFF303099 measured in PBS at 28 °C was comparable to that of NZP2213 (60.28 % ± 2.8), but it declined ca. 2.5-fold in the PYG medium. A much lower number of bacteria interacted with *A. castellanii* at 18 °C. In the same medium (PBS) at 28 °C, the recovery of strains NZP2213 and MAFF303099 from amoebae cells decreased ca. 5- and 4-fold, respectively. On the other hand, in the nutrient-rich medium PYG at 18 °C, the number of bacteria engulfed by the amoebae was statistically insignificant.

**Fig. 2:**
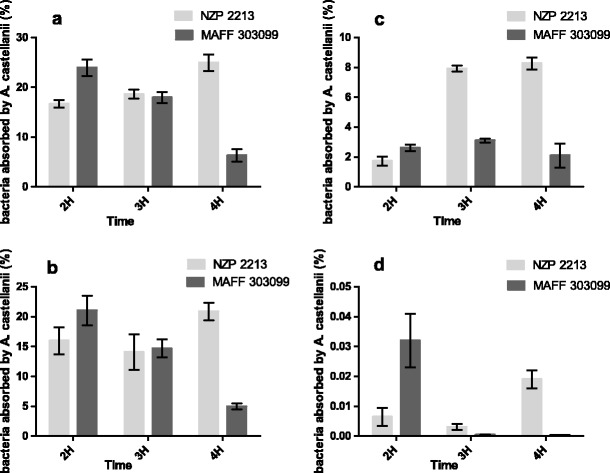
Uptake rates of *A. castellanii* Neff for *Mesorhizobium* spp. presented as a percentage of bacterial inocula (CFU = 6 × 10^6^). Experiment carried out **a** in PBS at 28 °C, **b** in PYG at 28 °C, **c** in PBS at 18 °C and **d** in PYG at 18 °C. Results are the mean of three independent experiments performed in duplicate. *Error bars* represent standard deviation (*n* = 3)

**Fig. 3:**
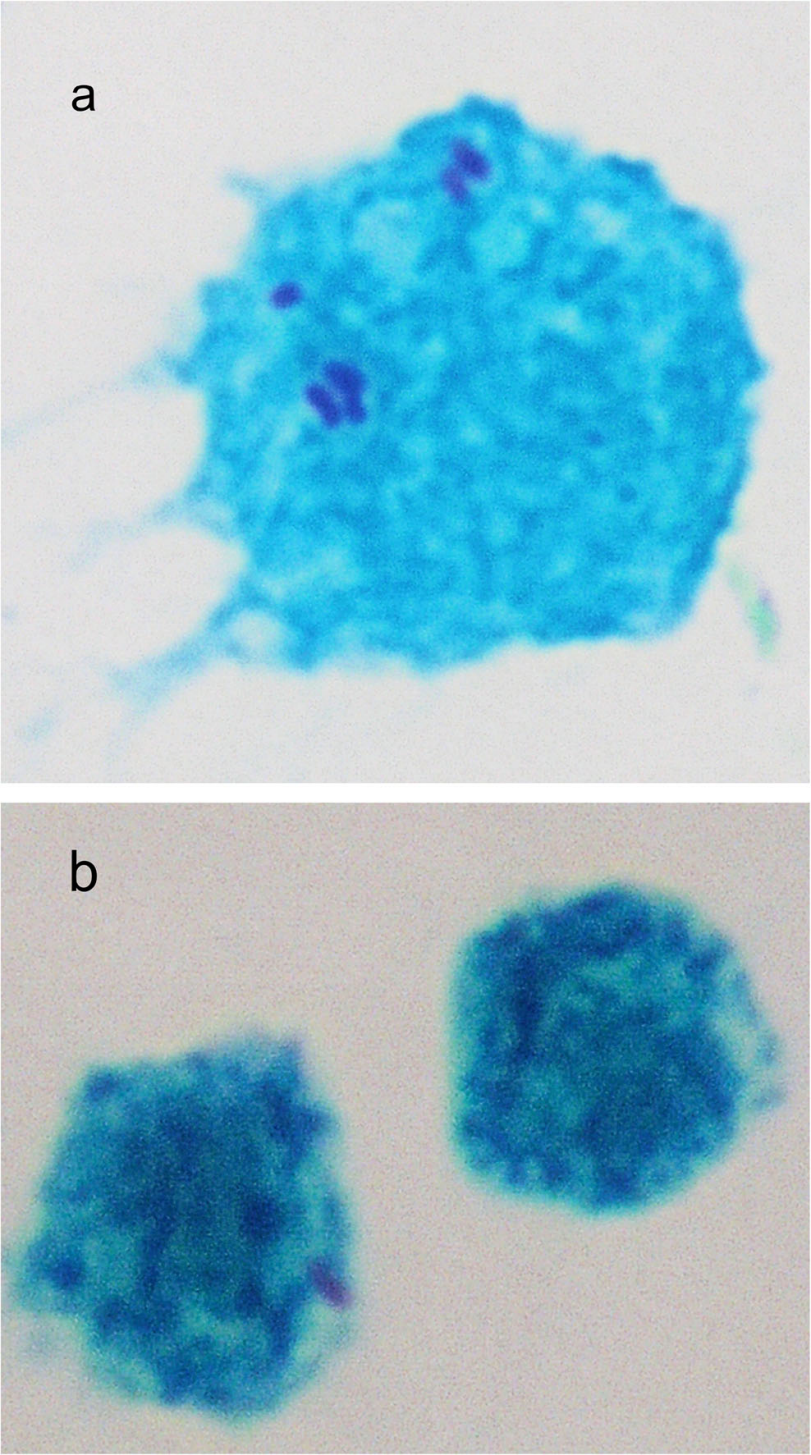
*M. loti* NZP2213 (*purple-stained rods*) detected inside *A. castellanii* (*green-stained cell*) after 4 h (**a**) and 6 h (**b**) coculture using Gimenez staining

The fate of the bacteria inside amoeba cells was tested using the antibiotic protection assay. The results are presented in Fig. 2. The bacterial uptake at 28 °C after 2- and 3-h co-incubation was almost constant (Fig. 2a, b). However, the amount of the NZP2213 bacteria residing inside the amoebae increased significantly after 4-h co-incubation, while the number of MAFF303099 cells was reduced. The percentage of the initial inoculum of these strains in PBS and in PYG reached 24.94 % ± 1.67 and 6.28 % ± 1.25 (Fig. 2a) and 20.86 % ± 1.5 and 4.94 % ± 0.47 (Fig. 2b), respectively. The rate of bacterial uptake measured at 18 °C in PBS was similar, indicating that the uptake rate of strain NZP2213 increased and that of strain MAFF303099 considerably decreased along with the co-incubation time (Fig. 2c). On the basis of the results obtained, it can be deduced that the increase in the number of NZP2213 bacteria residing in the amoebas was due to both their active uptake and multiplication in the course of incubation (Fig. 3). The decline in the number of viable MAFF303099 cells inside *Acanthamoeba* may indicate that with time amoebae utilize these bacteria as a source of nutrients. The intracellular survival assays were performed in order to confirm this suggestion. In that experiment, NZP2213 bacteria that were not taken up after 2-h co-incubation with amoebae were killed with antibiotics, while those inside the protists were left for 24 h. Extended residence time of the bacteria inside the amoebae was applied to show whether they are digested by amoebae or can survive inside. After the incubation, the bacteria were seeded on TY agar plates without prior dilution. The abundance of strain NZP2213 colonies was high, whereas that of strain MAFF303099 was negligible.

Consequently, the capability of the NZP2213 bacteria to self-release from the host cells and temperature-dependence of the process were determined. Since the highest number of NZP2213 bacteria resided in the amoebae after the 4-h co-incubation, that time was set in the experiments. The test was carried out in the same way as the invasion assays except amoeba lysis prior to bacteria seeding on TY plates. No bacteria colonies were seen when temperature 37 °C was applied. On the contrary, the yield of bacteria was abundant at 28 °C after 24 h of appearance of the colonies. When TY plates were incubated at 18 °C, no colonies appeared before day 3, which suggests prolongation of the lag phase of the mesorhizobia released at the lower temperature.

### Mode of *Mesorhizobium* Uptake

As known, *A. castellanii* utilizes a diverse repertoire of extracellular receptors involved in the intercellular communication. The endocytosis of *Legionella pneumophila* by *A. castellanii* is mediated by C-type lectin, i.e. mannose-binding protein (MBP). Lectins recognizing other sugars (i.e. D-galcatoside/L-rhamnose or N-acetyl-D-galactosamine) were also reported [35]. The pretreatment of amoeba cells with D-mannose, D-galactose or D-glucose was performed to determine if any of them blocks adhesion of *Mesorhizobium* bacteria to amoeba cells.

**Fig. 4:**
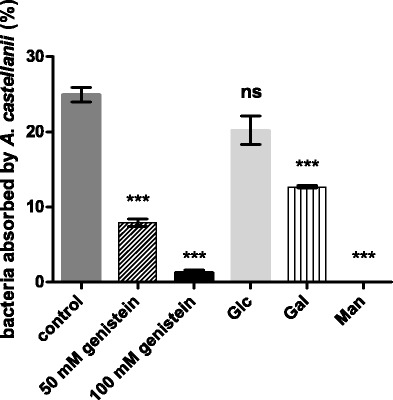
Results of phagocytosis inhibitory assays performed with use of different exogenous sugars (Glc, Gal, Man; at a final concentration 100 mM in PBS) and genistein (a protein tyrosine kinase inhibitor). These experiments were performed in PBS medium at 28 °C for 4 h and bacteria internalized by amoeba without an inhibitory treatment were assumed as a control. The *bars* labelled with stars represent the percent of viable intra-amoebic bacteria, which are significantly different at *p* < 0.001, and the *extended bars* represent the standard deviation (*n* = 3). All *p* values have been corrected for multiple comparisons by multiplying the calculated *p* value by the number of comparisons made (Tukey’s test through GraphPad Prism 6.0 software)

Since only NZP2213 was able to survive inside the amoebae, the strain was chosen for these studies. The most efficient conditions established for the invasive assay (28 °C, PBS medium and 4-h co-incubation) were applied. Our findings demonstrated that the exposure of amoebae to D-mannose at a 100-mM concentration completely reduced the attachment of bacteria, which suggests involvement of mannose-binding lectin in the uptake of bacteria by amoeba (Fig. 4). The pretreatment of *A. castellanii* with D-galactose reduced by half the effect of the bacterial uptake when compared to the amount of bacteria recovered from the untreated amoebae (Fig. 4). In turn, the pretreatment of *A. castellanii* with D-glucose exerted a very weak inhibitory effect on bacterial attachment (Fig. 4).

The main role of MBP in endocytosis is well documented, and it is known that pathogenic *A. castellanii* strains produce robust amounts of MBP [7]. We decided to examine *Acanthamoeba* strain AcP3 isolated from a keratitis patient assuming that the more MBP on the surface of amoebae, the higher number of adherent and intracellular NZP2213 bacteria should be detected. The results of the association assays showed that 87.35 % ± 6.15 of the initial bacterial inoculum adhere to AcP3 in comparison to 74.44 % ± 3.9 in the case of the nonpathogenic strain *A. castellanii*. Also, the ratio of associated bacteria per amoeba was higher in the case of AcP3 than in *A. castellanii* and reached 17.25 and 10.695, respectively. The amount of engulfed bacteria was also higher when the NZP2213 bacteria were co-incubated with the strain AcP3 than with *A. castellanii* and accounted for 66.5 % ± 12 (*p* < 0.05) and 24.94 % ± 1.67, respectively. The association and the invasion assays indicated that the NZP2213 bacteria in cocultures with strain AcP3 resulted in greater numbers of adherent and intracellular bacteria than in the incubation with *A. castellanii*. The combined results indicate an essential role of D-mannose-binding lectins in attachment of the NZP2213 bacteria to amoebae. A minor contribution of D-galactose-dependent lectins in this process was observed (Fig. 4).

To study the involvement of an intracellular signalling pathway, the invasion assays were performed in the presence of genistein, a natural protein tyrosine kinase inhibitor. The pretreatment of the amoebae with genistein at different concentrations inhibited significantly the uptake of the NZP2213 bacteria by the amoebae. The decrease in the percentage of the bacteria remaining inside was 68.4 and 95 % (*p* < 0.001) at 50 and 100 μM of geneistein, respectively (Fig. 4). These results suggest that protein tyrosine kinases could be triggered in engulfment of *M. loti* NZP2213 by *A. castellanii*.

### Growth in Amoebae Affects *Mesorhizobium* NZP2213 Capability to Infect Legume Host Plant

**Fig. 5:**
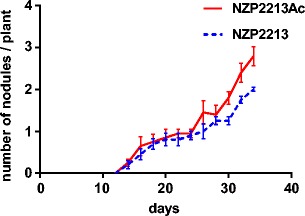
Nodule formation kinetics of the *M. loti* NZP2213 wild strain and NZP2213 released from *A. castellanii* (NZP2213Ac) determined as the number of nodules per plant. Each *point* represents the mean ± standard error of determinations in three independent sets of 20 plants

*Acanthamoeba* spp. can be isolated from the rhizosphere of many plants including the *Leguminosae* family [36]. A great number of molecular mechanisms mediating the communication of partners in symbiosis and pathogenesis are quite similar [37]. The growth of bacteria inside amoebae could change their physiological properties [15–18]. The influence of facultative survival of symbiotic bacteria from the genus *Mesorhizobium* inside amoebae on their symbiotic properties was examined. In the studies, the ARB-type strain *M. loti* NZP2213 was used, which forms functional nitrogen-fixing nodules (Nod^+^Fix^+^) on *L. corniculatus* [38]. The ability of strain NZP2213 and NZP2213 released from *A. castellanii* (described hereafter as NZP2213Ac) to form nodules on *Lotus* plants was compared. The results obtained indicated that the fresh mass of *L. corniculatus* shoots inoculated either with the NZP2213 or NZP2213Ac bacteria was quite similar (35 and 27 mg/plant, respectively) and two times higher than that of uninoculated plants (15 mg/plant). The fresh mass of roots infected with NZP2213 and NZP2213Ac was also similar, 30 and 27 mg per plant, respectively, and three times higher than that of root mass of noninfected plants (11 mg/plant). The examination of the nodulation ability showed that the number of nodules induced by the native bacteria after approximately a month was two per plant, while the number of nodules induced by bacteria released from amoebae was three per plant (Fig. 5). The ratio of the fresh mass of shoots and roots of plants inoculated with NZP221 and NZP2213Ac was 1.16:1 and 1:1, respectively, indicating that *Mesorhizobium* released from *A. castellanii* exhibited slightly diminished nitrogen fixation efficiency, while the nodule-forming capacity was increased.

## Discussion

Birdsfoot trefoil (*Lotus corniculatus* L.) (BFT) is a legume grown mainly for green fodder production in temperate regions. It is a high-quality forage rich in protein, minerals and carotene, which can be grazed or cut for hay or silage. The forage legume birdsfoot trefoil supports reduction of soil and water acidification and enteric methane production. Furthermore, the economic aspects of BFT grazing-based systems should be mentioned: (i) reduced feed costs and energy costs, (ii) increased meat and milk production and (iii) reducing dairy cow mortality through suppression of internal parasites [39]. An additional advantage of this plant is related to its ability to accumulate nitrogen-fixing bacteria [24]. Among legume plants, birdsfoot trefoil is better adapted to soils that are poorly drained, droughty or of low fertility [40]. It is also known that unlike alfalfa (*Medicago sativa* L.) and most true clovers (*Trifolium* sp.), the tannin content of BFT allows it to be grazed, even in pure stands, without risk of bloat. Birdsfoot trefoil has reduced dairy manure ammonia emissions compared with alfalfa, diminished urine nitrogen content and decreased mineralization of nitrogen from faeces compared with white clover (*Trifolium repens* L.), which lowers the potential for nitrate leaching. Besides these advantages, it is also helpful for erosion control, re-vegetation, wildlife habitat, improved soil pH, drainage and fertility [39].

The interactions between the microsymbiont and the host plant during nodule formation could be influenced by various environmental factors. The features that may affect the fitness of both partners such as competitiveness of the microsymbiont are extensively studied [41]. To the best of our knowledge, there are no many data concerning the effect of the presence of commonly occurring protozoa and microsymbiont abundance [5, 6]. On the other hand, the presence of grazing-resistant bacteria has also been reported. The association between FLA and pathogenic bacteria is better understood [7] although some reports describe AAM or ARB rhizobia [10–13]. It seems reasonable to study the relationships between rhizobia and amoebae in terms of a possible impact on the symbiotic properties of bacteria [37, 42, 43].

Since *M. huakuii* subsp. *rengei* was determined to coexist with *M. loti* strains in the rhizosphere of particular plant host [26], strains *M. loti* NZP2213 and *M. huakuii* sbv. *loti* MAFF303099 capable of formation of nitrogen-fixing nodules on *L. corniculatus* were tested [26, 27]. Both species form different lineages [33], although they are closely related and produce a similar profile of cellular fatty acids [44]. Their lipid A also possesses a similar structure and contains long-chain 27-hydroxyoctacosanoic acid (27-OH-28:0) [45–47]. The occurrence of the 27-OH-28:0 acid has been demonstrated in a variety of bacteria that survive within intracellular host membrane-derived compartments [47]. Similar structural features of lipid A were identified in the LPS of *Legionella* spp. [48], an endosymbiont of *Acanthamoeba* spp. The main difference between both tested strains is that they possess different secretion systems, i.e. T3SS and T4SS in MAFF303099 and NZP2213, respectively [22]. In this paper, differences between two strains of *Mesorhizobium* in their interactions with *Acanthamoeba* and the impact of the effectiveness of *L. corniculatus* nodulation have been demonstrated.

The results obtained indicated that the efficiency of association of the *Mesorhizobium* strains with amoebae was similar in all the tested conditions (Fig. 1). Thus, the association stage seems to be nonspecific. Since both tested bacterial strains have a similar structure of external polysaccharide-containing polymers [45–47], it is possible that they have similar epitopes responsible for the first interaction with a host. One of the candidates responsible for the nonspecific rhizobium-binding function seems to be the protein containing a legume lectin beta domain encoded in the *A. castellanii* Neff strain by gene ORF ACA1 1385100 [35]. The first step of adhesion of rhizobium to the roots of legume plants is also nonspecific. The 4-h co-incubation resulted in considerable reduction of the uptake of strain MAFF303099 by amoebae and increased uptake of NZP2213 (Fig. 2). Also, after prolonged time of co-incubation (24 h), the recovery of NZP2213 bacteria on TY plates was abundant, while no growth of MAFF303099 was detected. The above results may indicate that bacteria NZP2213 are internalized but sustain the amoeba defence system, while bacteria MAFF303099 undergo the endocytic degradation pathway. Since rhizobial proteins secreted by T3SS are involved in suppressing or activating defence responses in the early stages of infection of some plant hosts and T4SS of *M. loti* R7A (closely related to NZP2213) is involved in the establishment of symbiosis, acting in a host-specific manner to either assist (*L. corniculatus*) or impede nodulation (*Leucaena leucocephala*) [22], it can be assumed that T3SS in MAFF303099 and T4SS in NZP2213 may play a role in the interactions with *A. castellanii*. Many Gram-negative bacterial intracellular pathogens (*Francisella tularensis*, *L. pneumophila*) use T4SS to multiply symbiotically within amoebae [20, 21]. The results obtained have shown that NZP2213 inside amoeba cells persist as those pathogens, indicating that the components of T4SS play a role in the endosymbiotic interaction. Other animal and plant pathogens utilize T3SS to allow persistence within host cells [20, 49]; therefore, it can be expected that MAFF303099 would sustain inside amoeba. Contrary to our expectations, the fate of MAFF303099 inside *A. castellanii* did not resemble the fate of intracellular pathogens expressing T3SS (*Escherichia coli* K1) [20]. It is known that in plant systems, most of the T3SS genes of the MAFF303099 strain are expressed in the presence of flavonoid inducers [49]. Probably, in our experiments, no or only few effector proteins were excreted thus MAFF303099 behaved as an extracellular nonpathogenic and T3SS-negative *E. coli* K12 strain ingested as a food by amoeba [23]. Taking into account these observations, further studies are necessary to explain the T3SS function in interactions with amoeba.

Since temperature influences the outcome of amoeba infection [33, 50], the interactions were tested at 28 °C, a temperature required for optimal growth of both bacteria and amoebae, and 18 °C, related to soil conditions. The association and the uptake rate of bacteria at 28 °C were similar until 3 h postinfection regardless of the medium (PBS or PYG). Contrarily, at 18 °C, the mesorhizobia were more effectively taken up the by amoebae in the PBS buffer than in the nutrient-rich PYG medium. Since phagocytosis of bacterial particles is energy consuming, in PYG, amoebae preferentially utilize the compounds of the culture medium.

It was demonstrated by other authors that the uptake of endosymbionts by *Acanthamoeba* in a receptor-dependent phagocytosis pathway is mediated mainly by mannose-binding protein [51, 52]. The association assays with exogenously applied sugars showed that the pre-incubation of amoeba cells with D-mannose completely blocked the uptake of the NZP2213 bacteria, while in the presence of D-galactose, only half of viable bacteria could be recovered (Fig. 4). The results obtained suggest that D-mannose- and, to some extent, D-galactose-dependent receptors are involved in adhesion of *Mesorhizobium*. In the presence of genistein, a natural inhibitor of protein tyrosine kinase, a significant decrease in the uptake of the NZP2213 bacteria by amoebae (reduction up to 95 % at a 100-μM concentration) was found (Fig. 4). We can presume that a tyrosine kinase-mediated actin polymerization signal is involved in phagocytosis of mesorhizobia by *A. castellanii*, as it was demonstrated in the case of *E. coli* [51].

Although *L. corniculatus* seems to grow well in most environments, the adoption of this legume is limited due to its slow establishment and poor persistence in some climates. To improve the process, rhizobial inoculation of seeds by effective, specific strains is needed when sowing, particularly on land where birdsfoot trefoil has not been cultivated before. The development of rhizobia inoculants can involve either the selection or the improvement of strains in order to achieve high symbiotic effectiveness and tolerance to environmental conditions. Competitiveness for nodulation is a desirable trait in rhizobial inoculant strains as well. There are numerous reports concerning the efficacy of rhizobial inoculants to form nitrogen-fixing root nodules on legume crop plants. The reports of other authors [10–13] and the results of the present studies indicate that some rhizobial strains are able to survive the engulfment by amoebae and to self-release. It is also known that the intracellular maintenance of bacteria in *Acanthamoeba* often enhances the virulence capabilities of endosymbionts [16]. The results of studies of the infection capabilities of the NZP2213 bacteria that survived inside the amoebae indicated an increase in the potential of mesorhizobia to infect the host plant *L. corniculatus* (Fig. 5). Since, as mentioned before, the effector proteins of T4SS favour the growth of *M. loti* R7A in *L. corniculatus* [22], the expression of T4SS genes in the ARB *Mesorhizobium* strain (NZP2213) is increased, which could be confirmed by a gene expression assay.

Another advantage of the capability of some *Mesorhizobium* strains to survive inside amoebae could be the possibility to hide from unfavourable environmental conditions, e.g. the deleterious effects of antimicrobial compounds released by other bacteria [53, 54].
